# Editorial: Hunting for inflammation mediators: identifying novel biomarkers for autoimmune and autoinflammatory diseases

**DOI:** 10.3389/fimmu.2026.1778299

**Published:** 2026-01-13

**Authors:** Li Zeng, Yu Fang, Shintaro Horie, Jian Liu

**Affiliations:** 1 Shanxi Academy of Medical Sciences, Taiyuan, China; 2 Centre for Clinical Laboratories, Affiliated Hospital of Guizhou Medical University, Guiyang, Guizhou, China; 3 Department of Ophthalmology and Visual Science, Institute of Science Tokyo, Tokyo, Japan; 4 Academic Unit of Ophthalmology, School of Biochemistry and Biomedical Sciences, Faculty of Health and Life Sciences, University of Bristol, Bristol, United Kingdom

**Keywords:** autoimmune, autoinflammation, biomarkers, inflammation, inflammation mediators

Autoimmune and autoinflammatory diseases are the costly trade-offs of a highly sophisticated and adaptable immune system. In the shared effort to identify biomarkers, sixteen studies spanning diverse disease types and employing both modern computational approaches and classical wet-lab techniques have converged. This Research Topic comprises ten original research articles, two brief research reports, one case report, two reviews, and one systematic review.

The active role of platelets in immune-mediated inflammation, *via* soluble cytokines and surface glycoproteins, has gained increasing recognition (1). In a retrospective study of 1,002 critically ill patients with rheumatic heart disease (RHD), Zhang and Ni analysed systemic inflammatory biomarkers using the Medical Information Mart for Intensive Care IV (MIMIC-IV) database. A higher platelet-to-lymphocyte ratio (PLR), reflecting elevated platelet counts and reduced lymphocyte counts, was most strongly associated with increased mortality risk and demonstrated a robust linear relationship with 30-day all-cause mortality, independent of demographics, disease severity, and comorbidities including respiratory failure, diabetes, renal disease or sepsis. These findings support PLR as an accessible and independent prognostic marker for early risk stratification in critically ill RHD patients. In a separate analysis, Xiong and Yu identified the neutrophil-to-albumin ratio (NPAR) and neutrophil-to-lymphocyte ratio (NLR) as positively correlated with psoriasis prevalence among adults from the National Health and Nutrition Examination Survey (NHANES). Using bulk transcriptomic analysis and computational algorithms, Ma et al. unravelled the inflammatory basis of dysthyroid optic neuropathy (DON), a severe manifestation of Graves’ hyperthyroidism orbitopathy. They identified key differentially expressed genes (DEGs) involved in heightened immune responses, inflammation, and fibrotic remodelling in retro-orbital adipose tissues from DON patients, compared to individuals with non-DON thyroid eye diseases and healthy controls. Notably, inflammation-related DEGs such as *CCL1*, *SLCO2A1* and *HP* were validated by qRT-PCR, supporting their potential diagnostic and therapeutic relevance.

Liu et al. addressed the long-standing challenge of distinguishing lupus nephritis (LN) from non-renal SLE. Although confirming serum CXCL13 as a robust SLE biomarker, they revealed its limited specificity for LN. Their key mechanistic insight was the identification of reduced circulating CXCR5^+^ T cells, particularly the CD4^+^ subset, as a superior discriminative marker. This finding was linked to a pathogenic redistribution mechanism in which elevated renal CXCL13 recruits these cells from the circulation to inflamed kidney tissue. This work highlights circulating CXCR5^+^ T cells as a non-invasive biomarker of renal involvement in SLE.

Cystatin C (Cys-C), a cysteine protease inhibitor that controls the turnover of multifunctional enzymes involved in immunological processes, has been implicated in inflammatory autoimmunity (2). Consistent with this, He et al. reported that higher Cys-C levels were associated with disease severity and a Th2-to-Th1 shift in acetylcholine receptor antibody-positive myasthenia gravis (MG), based on data from UK Biobank and Chinese cohorts. Ethnic differences can significantly influence the prevalence and seropositivity of autoantibodies in autoimmune diseases, particularly myositis-specific autoantibodies (MSAs) (3). In a retrospective study of a Taiwanese cohort, Hsiao et al. found that strong positivity for antibodies against small ubiquitin-like modifier 1-activating enzyme subunit 1 (SAE1), a member of the MSAs, was closely associated with idiopathic inflammatory myopathy (IIM) and interstitial lung disease (ILD). The high diagnostic performance of SAE1 antibodies measured by line immunoassay (LIA) underlines the importance of early ILD screening and close monitoring in these patients. Zhu et al. elucidated a previously underappreciated pathogenic inflammatory loop in granulomatous lobular mastitis (GLM), identifying the alarmin S100A8/S100A9 as a critical driver of neutrophil extracellular trap (NET) formation *via* direct interaction with and activation of peptidylarginine deiminase 4 (PAD4). The therapeutic relevance of this axis was shown by paquinimod-mediated inhibition of S100A8/S100A9 in a mouse GLM model.

Corticosteroids (CS) are a mainstay in the treatment of non-infectious uveitis, yet a considerable proportion of patients either fail to respond adequately or develop adverse effects that necessitate discontinuation of therapy (4). To identify predictors of CS response, Valenzuela et al. investigated the dynamic expression of glucocorticoid receptor alpha (GRα) and cytokine profiles in peripheral blood mononuclear cells from 19 patients. Within two weeks of CS initiation, CS-sensitive patients exhibited greater increases in IL-10 levels and in the IL-10/IL-6, IL-10/IL-17A, and IL-10/GRα ratios compared to CS-refractory individuals. Among these, IL-10 expression and IL-6/IL-10 ratio demonstrated the strongest predictive value for identifying CS refractoriness.

Beyond cytokines and immune cells, other circulating components have emerged as informative biomarkers. Buttari et al. demonstrated that extracellular microvesicles (EMVs) isolated from the plasma of patients with rheumatoid arthritis (RA) promoted allostimulatory maturation of naïve dendritic cells (DCs), enhanced cytokine secretion, and increased T-cell stimulatory capacity. Post-translational modifications (PTMs), particularly citrullination and carbamylation, within RA-derived EMVs, likely contribute to pathogenic immune modulation. In a case-control study, Vakrakou et al. proposed cerebrospinal fluid (CSF) biopsy as a minimally invasive tool for the early differentiation of paraneoplastic cases in autoimmune encephalitis (AEs). High-risk paraneoplastic antibodies (Kelch-11, Yo, Hu) were associated with increased cf-DNA integrity, indicative of necrotic cell death, while the complement split product C4d best discriminated intracellular from extracellular antibody-associated AEs. Elevated cf-DNA and C4d demonstrated high diagnostic accuracy, and the lack of correlation between CSF and plasma levels suggested a compartmentalised CNS immune response. As markers of immune activation and underlying endothelial damage in autoimmune diseases, circulating cell adhesion molecules (CAMs) are involved in the migration and adhesion of immune cells, with their binding to glycosylated ligands mediating leukocyte tethering and rolling along the endothelial surface. In a meta-analysis of 26 eligible studies, Przęczek et al. evaluated serum levels of intercellular CAM-1 (sICAM-1), vascular CAM-1(sVCAM-1), sE-selectin, sP-selectin, and sL-selectin in patients with inflammatory bowel disease (IBD). Both sICAM-1 and sE-selectin were significantly elevated in IBD patients compared to controls, but only sICAM-1 was significantly higher in Crohn’s disease (CD) *vs* ulcerative colitis (UC), and in active *vs* inactive disease groups, mirroring observations in rheumatoid arthritis (RA) (5) and systemic sclerosis (6). Other CAMs showed nonsignificant trends toward elevation, which may be attributable to the smaller number of available studies. Despite limitations in specificity, CAMs may serve as adjunct biomarkers for monitoring inflammation activity rather than standalone diagnostic tools.

The derangement of immune responses and uncontrolled inflammation are often intertwined with disruptions in other biological processes, such as sleep disturbance (7) and viral infection (8). Uncovering the underlying interactions may help identify druggable targets for therapeutic intervention. Wu et al. analysed Gene Expression Omnibus (GEO) datasets using bioinformatics tools and identified shared viral response-related genes and pathways in patients with insomnia and autoimmune uveitis (AU), suggesting a potential long-term and bidirectional etiological interaction among viral infection, insomnia and AU. Specifically, the interferon regulators *IFI44* and *IRF9* were proposed as diagnostic biomarkers for insomnia-associated AU. Radhakrishna et al. revealed epigenetically dysregulated genes related to circadian clock circuitry in peripheral blood specimens from patients with hidradenitis suppurativa (HS). A panel of differentially methylated genes exhibiting 8-, 12-, or 24-hour oscillatory periodicity was identified, impacting key pathways involving MAPK, WNT signalling, xenobiotic metabolism, oxidative stress, hypoxia, protein folding, DNA repair, cell survival, and, notably, hair and skin function.

Liu and Li reported a rare adult case of myelin oligodendrocyte glycoprotein-associated cerebral cortical encephalitis (MOG-CCE), a neuroinflammatory entity within the spectrum of MOG antibody-associated autoimmune disease (MOGAD), first described in 2017. This case highlights the importance of timely and targeted immunotherapy guided by a precise diagnosis integrating clinical presentations, laboratory testing, neuroimaging, and pathological findings. A live cell-based assay was emphasised for accurate titration of MOG-IgG owing to its superior sensitivity and specificity. The generation of MOG antibodies in this patient was suspected to have been triggered by a preceding Epstein-Barr virus (EBV) infection. Differential diagnosis included other causes of encephalitis with overlapping features, such as anti-N-methyl-D-aspartate receptor (NMDAR) encephalitis and herpes simplex virus encephalitis (HSVE), based on clinical, imaging and serological features. Maintenance immunotherapy for ≥3 months was recommended to reduce relapse risk. This case illustrates the need for heightened clinical awareness and further refinement of the disease course characterisation, treatment responses, and long-term prognosis in MOG-CCE.

Finally, a review by Fan et al. discussed the diagnostic and prognostic potential of elevated HMGB1 protein levels in blood and tissues for autoimmune skin diseases, including vitiligo, psoriasis, atopic dermatitis, alopecia areata, polymyositis, dermatomyositis and pemphigus. Despite its promise as a biomarker, challenges remain in developing HMGB1-targeted therapies due to its dynamic and context-dependent roles, redox status, isoform variability, and widespread expression across multiple disease states. In a comprehensive review, Liu et al. challenged the long-held concept of cochlear immune privilege by synthesising major advances in cochlear immunology from 2020 to 2025. The review systematically covers the components of the cochlear immune system and current therapeutic options for hearing disorders, including both traditional immunosuppressants and emerging immunomodulatory molecules. Looking ahead, the identification of reliable biomarkers, clarification of immune cell heterogeneity, optimization of drug delivery, and development of targeted immunomodulatory strategies are emphasized as critical directions.

This modest yet diverse Research Topic of biomarker studies has concluded, but our curiosity persists, and the quest continues.

## References

[1] Scherlinger M Richez C Tsokos GC Boilard E Blanco P . The role of platelets in immune-mediated inflammatory diseases. Nat Rev Immunol. (2023) 23:495–510. doi: 10.1038/s41577-023-00834-4, PMID: 36707719 PMC9882748

[2] Zi M Xu Y . Involvement of cystatin C in immunity and apoptosis. Immunol Lett. (2018) 196:80–90. doi: 10.1016/j.imlet.2018.01.006, PMID: 29355583 PMC7112947

[3] Beam MJ Montgomery A Anastasiou C Schmajuk G . Association between self-reported race and ethnicity and myositis-specific autoantibodies in a diverse cohort of patients with inflammatory myopathy. Clin Rheumatol. (2023) 42:3043–7. doi: 10.1007/s10067-023-06719-0, PMID: 37542130 PMC10587270

[4] Chang YC Kao TE Chen CL Lin YC Hwang DK Hwang YS . Use of corticosteroids in non-infectious uveitis - expert consensus in Taiwan. Ann Med. (2024) 56:2352019. doi: 10.1080/07853890.2024.2352019, PMID: 38747459 PMC11097703

[5] Mangoni AA Zinellu A . A systematic review and meta-analysis of circulating adhesion molecules in rheumatoid arthritis. Inflammation Res. (2024) 73:305–27. doi: 10.1007/s00011-023-01837-6, PMID: 38240792 PMC10894129

[6] Mangoni AA Zinellu A . Circulating cell adhesion molecules in systemic sclerosis: a systematic review and meta-analysis. Front Immunol. (2024) 15:1438302. doi: 10.3389/fimmu.2024.1438302, PMID: 39234240 PMC11371573

[7] Stenger S Vorobyev A Bieber K Lange T Ludwig RJ Hundt JE . Insomnia increases the risk for specific autoimmune diseases: a large-scale retrospective cohort study. Front Netw Physiol. (2025) 5:1499297. doi: 10.3389/fnetp.2025.1499297, PMID: 40276126 PMC12018472

[8] Sokolovska L Cistjakovs M Matroze A Murovska M Sultanova A . From viral infection to autoimmune reaction: exploring the link between human herpesvirus 6 and autoimmune diseases. Microorganisms. (2024) 12:362. doi: 10.3390/microorganisms12020362, PMID: 38399766 PMC10892088

